# ToF performance evaluation of a PET insert with Digital Silicon Photomultiplier technology during MR operation

**DOI:** 10.1186/2197-7364-1-S1-A20

**Published:** 2014-07-29

**Authors:** David Schug, Jakob Wehner, Peter Michael Dueppenbecker, Bjoern Weissler, Pierre Gebhardt, Benjamin Goldschmidt, Torsten Solf, Volkmar Schulz

**Affiliations:** Department of Physics of Molecular Imaging Systems, Institute for Experimental Molecular Imaging, RWTH Aachen University, Aachen, Germany; Division of Imaging Sciences and Biomedical Engineering, King’s College London, London, UK; Philips Research Laboratories, Molecular Imaging Systems, Aachen, Germany; Institute for Experimental Molecular Imaging, RWTH Aachen University Hospital, Aachen, Germany; Philips Research, X-Ray Imaging Systems, Eindhoven, The Netherlands

2012, our group presented the Hyperion IID platform which uses PDPC’s digitial SiPMs (DPC). In this work we use the same platform equipped with scintillator dimensions closer to a clinical application. This allows an investigation of the time of flight (ToF) performance of the platform and its behavior during simultaneous MR operation.

We employ LYSO crystal arrays of 4×4×10 mm³ coupled to 4×4 PDPC DPC 3200-22 sensors allowing a one-to-one coupling of crystals to readout channels. Six sensor stacks are mounted onto a singles processing unit in a 2×3 arrangement. Two units are mounted on a gantry with a diameter of 216 mm. The DPCs are cooled down to approximately 5-10 °C under operation. We disable 20% of the worst cells and use an overvoltage (OV) of 2.0V and 2.5V.

To obtain the best time stamps we use the first photon trigger and employ data quality cuts to filter out crystal scatter events. A narrow energy window of 511±50 keV is used and a minimal light fraction of the main pixel of more than 65% is requested.

Using a Na22 point source in the isocenter of the modules the coincidence resolution time (CRT) of the two modules is evaluated outside the MR and inside the MR using different MR sequences. Gradient stress tests with switching z-gradients are performed.

Inside the B0 field at 2.0V overvoltage the energy resolution is 11.45% (FWHM) and the CRT is 250ps (FWHM). At 2.5V overvoltage, the energy resolution is 11.15% (FWHM) and the CRT is 240ps (FWHM). During the heavy z-gradient sequence the energy resolution is degraded by 4.1% at 2V and 9.2% at 2.5V. The degradation of the CRT is 25% at 2V and 52% at 2.5V OV. During standard TSE and EPI sequences the CRT and energy resolution is not influenced.

The Hyperion IID platform proofs to deliver good timing performance outside and inside an MR. The CRT is not influenced (<0.2%) using normal MR imaging sequences.

